# Supplementary material for: A 4-Week Diet Low or High in Advanced Glycation Endproducts Has Limited Impact on Gut Microbial Composition in Abdominally Obese Individuals: The deAGEing Trial
Source: Int J Mol Sci. 2022 May 10;23(10):5328. doi: 10.3390/ijms23105328 (PMC9141283; doi:10.3390/ijms23105328)
Supplement: Supplementary file 1 [file ijms-23-05328-s001.zip › ijms-1709998-supplementary.pdf]

Supplementary Figures

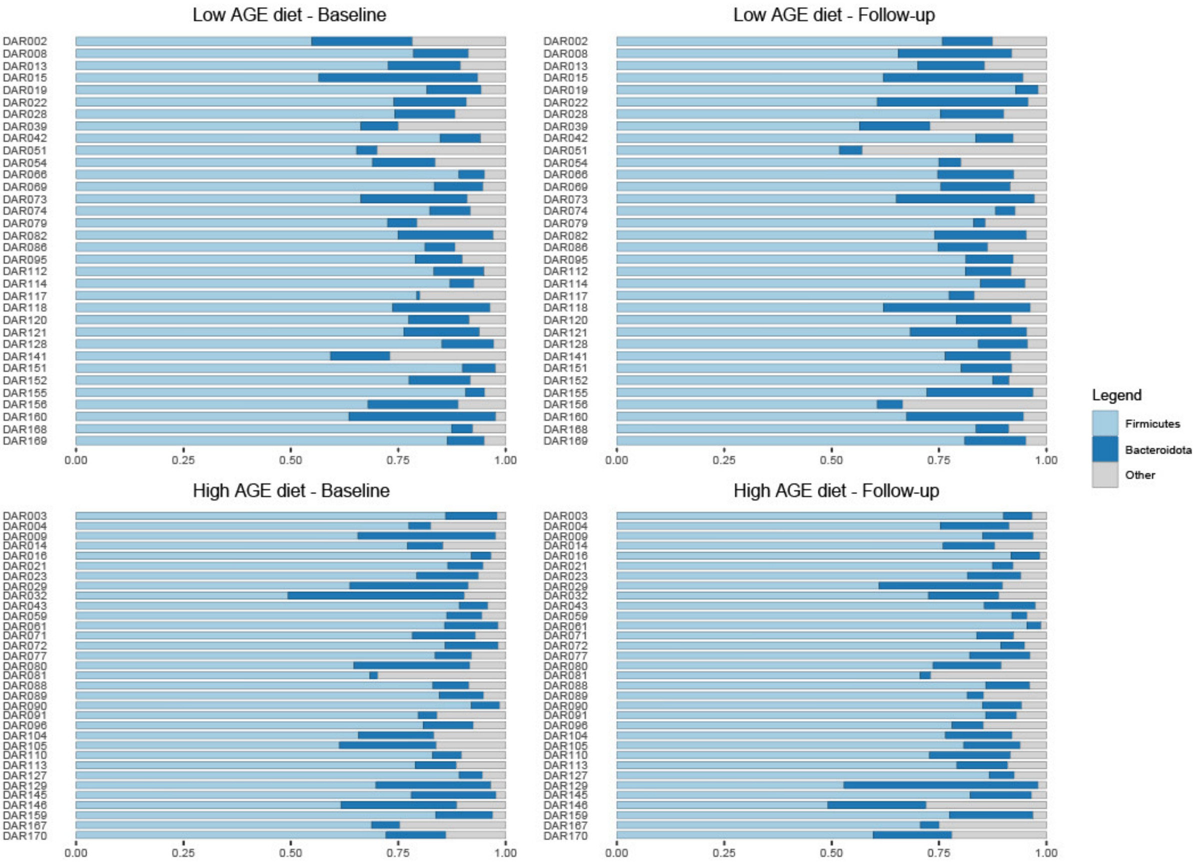

Supplemental Figure S1. Firmicutes/Bacteroidota ratio in abdominally obese individuals that finished a 4-week diet low or high in advanced glycation end products (*n*=70). "DAR" indicate unique participant identifiers (Dietary AGE Restriction).

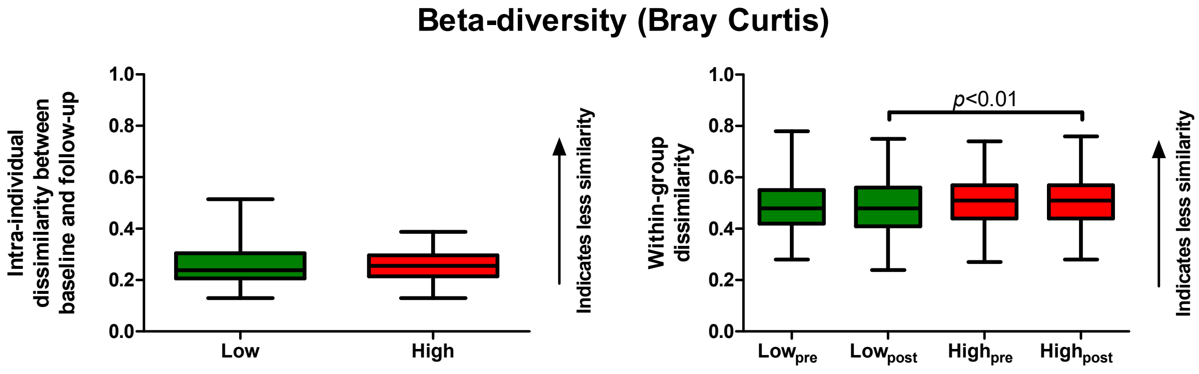

Supplemental Figure S2. Intra-individual (left) and within-group (right) Bray-Curtis dissimilarity after a 4-week diet low or high in advanced glycation endproducts in abdominally obese individuals (*n*=34 for the low AGE diet, *n*=36 for the high AGE diet).

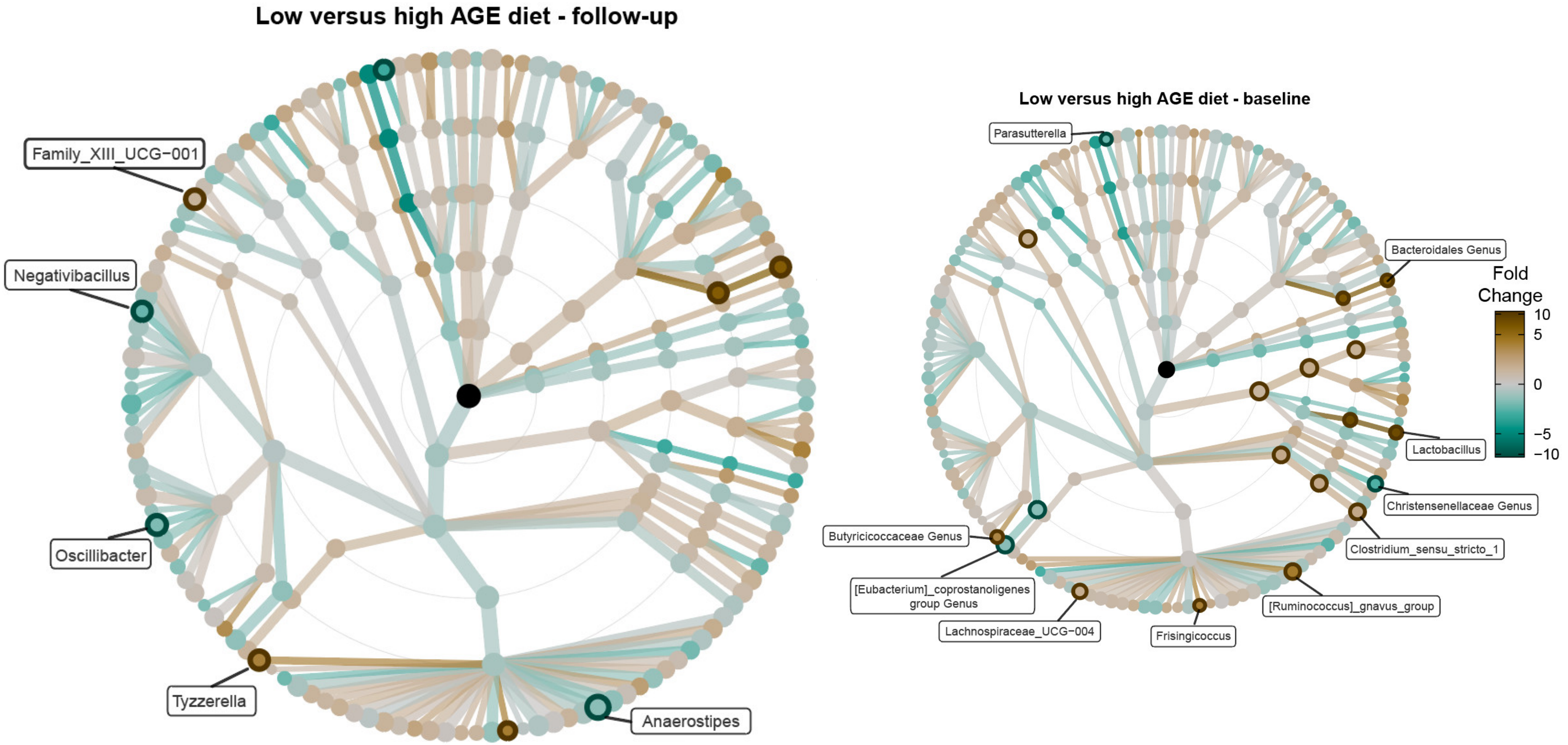

Supplemental Figure S3. Taxonomic association three showing differentially abundant taxa after a 4-week low (*n*=34) compared to the high AGE (*n* = 36) diet in abdominally obese individuals (left), and before allocation to the intervention diets (right). Statistical significance was assessed using beta binomial regression with additional adjustment for age and sex. Differentially abundant taxa are shown bold and labeled (before correction for multiple testing), and marked green (after correction for multiple testing).Taxa that were already differentially abundant at baseline were not labeled in the taxonomic association three after the intervention diets.

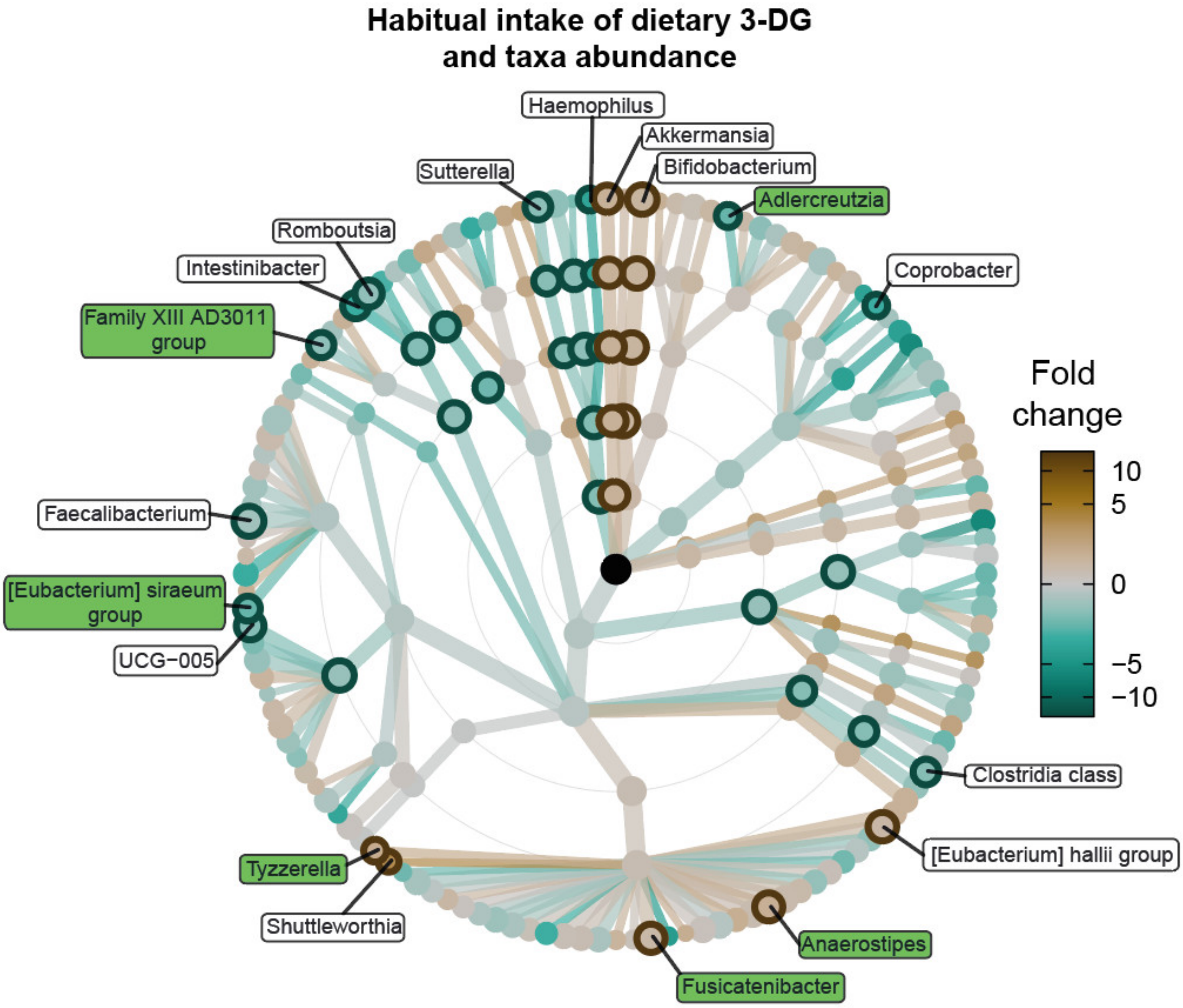

Supplemental Figure S4. Associations between habitual intake of 3-deoxyglucosone (3-DG, SD/day) and taxa abundance in 72 abdominally obese individuals. Statistical significance was assessed using beta binomial regression with adjustment for age, sex, energy intake, and the Dutch Healthy Diet index. Differentially abundant taxa are shown bold and labeled (before correction for multiple testing), and marked green (after correction for multiple testing).

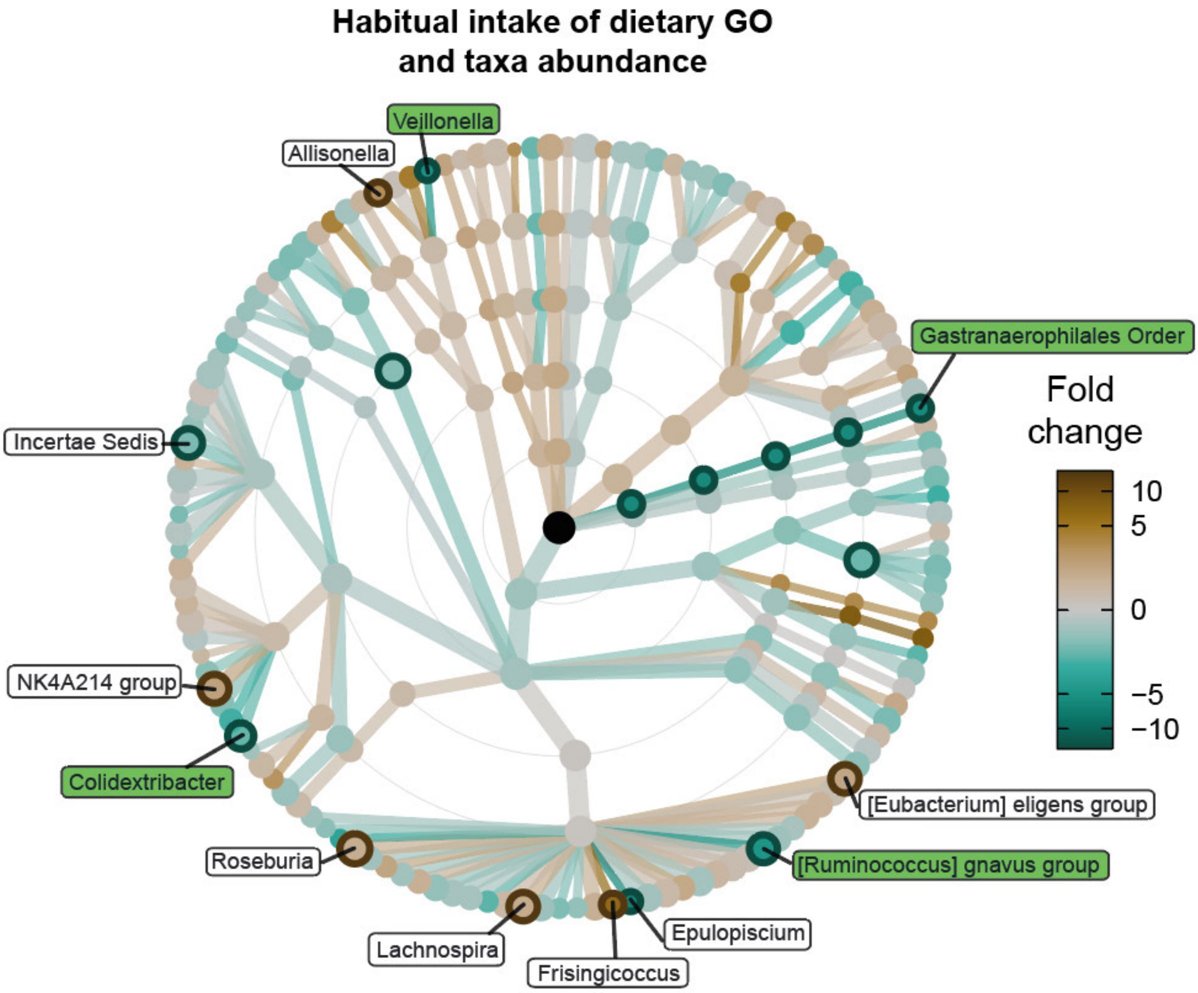

Supplemental Figure S5. Associations between habitual intake of glyoxal (GO, SD/day) and taxa abundance in 72 abdominally obese individuals. Statistical significance was assessed using beta binomial regression with adjustment for age, sex, energy intake, and the Dutch Healthy Diet index. Differentially abundant taxa are shown bold and labeled (before correction for multiple testing), and marked green (after correction for multiple testing).

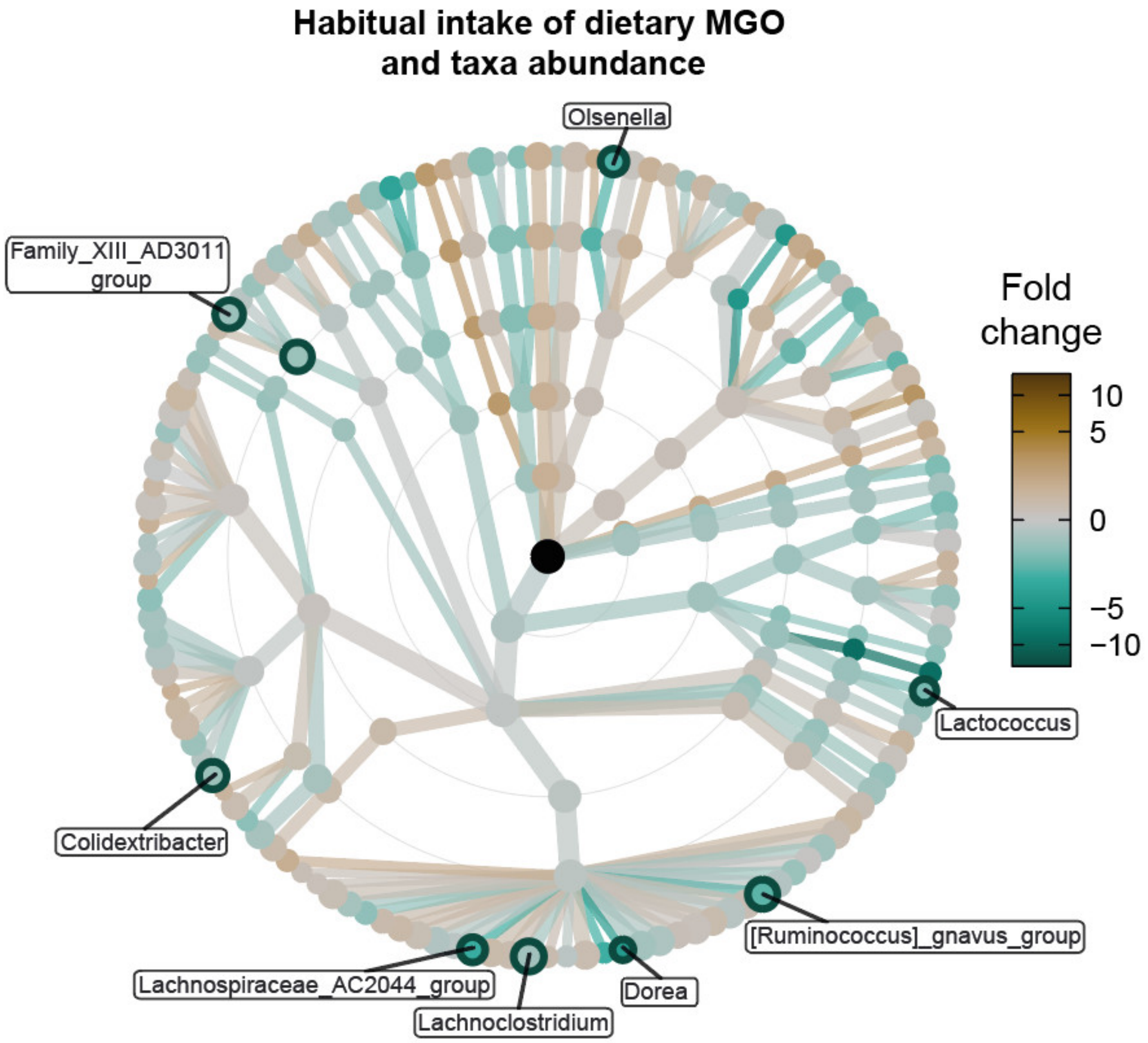

Supplemental Figure S6. Associations between habitual intake of methylglyoxal (MGO, SD/day) and taxa abundance in 72 abdominally obese individuals. Statistical significance was assessed using beta binomial regression with adjustment for age, sex, energy intake, and the Dutch Healthy Diet index. Differentially abundant taxa are shown bold and labeled (before correction for multiple testing), and marked green (after correction for multiple testing).

Supplementary Tables

Supplemental Table S1. Associations between habitual intake of dicarbonyls and alpha-diversity measures in abdominally obese individuals (*n* = 72).

	**Richness (total ASVs)** Beta (95% CI)	**Diversity (Shannon index)** Beta (95% CI)
**Dietary MGO**		
Crude model	1 (-7,9)	-0.01 (-0.08,0.06)
Model 1	-2 (-10,6)	-0.02 (-0.10,0.06)
Model 2	-3 (-16,10)	0.04 (-0.08,0.16)
**Dietary GO**		
Crude model	1 (-7,9)	-0.03 (-0.10,0.05)
Model 1	-2 (-10,6)	-0.03 (-0.11,0.04)
Model 2	0 (-19,19)	0.05 (-0.12,0.23)
**Dietary 3-DG (ln-transformed)**		
Crude model	-1 (-9,7)	-0.07 (-0.13,0.00)
Model 1	-2 (-10,6)	-0.07 (-0.14,0.01)
Model 2	-2 (-13,10)	-0.07 (-0.18,0.03)

Regression coefficients (standardized beta's and 95% CIs) represent the change in diversity or richness per 1 SD increase in daily MGO, GO, and 3-DG intake. Dietary 3-DG was log-transformed to attain a normal distribution. Abbreviations: 3-DG: 3-deoxyglucosone. GO: Glyoxal. MGO: Methylglyoxal.
Model 1: Adjusted for age and sex.
Model 2: Additionally adjusted for energy intake and the Dutch Healthy Diet Index.

Supplemental Table S2. Gut microbiota relative abundance in 72 abdominally obese individuals.

Genus	Relative abundance		
	Median	Quartile 1	Quartile 3
Blautia	7,26%	4,59%	9,95%
Faecalibacterium	6,61%	4,36%	9,16%
Ruminococcus	5,83%	1,99%	12,71%
Agathobacter	4,88%	2,49%	9,45%
Subdoligranulum	4,13%	2,76%	7,03%
Bacteroides	3,95%	2,54%	7,57%
Bifidobacterium	2,83%	0,71%	5,02%
Coprococcus	2,59%	1,47%	4,88%
Dorea	2,11%	1,42%	3,12%
[Eubacterium]_hallii_group	1,84%	1,19%	2,48%
Roseburia	1,59%	0,94%	2,41%
[Eubacterium]_coprostanoligenes_group Family	1,40%	0,79%	2,40%
UCG-002	1,32%	0,78%	1,94%
Fusicatenibacter	1,29%	0,74%	2,21%
[Ruminococcus]_torques_group	1,27%	0,84%	2,60%
Collinsella	1,17%	0,68%	1,51%
Anaerostipes	1,08%	0,50%	1,79%
Alistipes	0,81%	0,39%	1,34%
Streptococcus	0,79%	0,33%	1,96%
Christensenellaceae_R-7_group	0,78%	0,27%	1,88%
Parabacteroides	0,76%	0,41%	1,17%
UCG-005	0,75%	0,38%	1,20%
Lachnospiraceae Family	0,64%	0,49%	1,02%
Lachnospiraceae_ND3007_group	0,62%	0,34%	1,00%
Lachnospiraceae_NK4A136_group	0,57%	0,27%	1,16%
Lachnoclostridium	0,55%	0,40%	1,03%
[Ruminococcus]_gauvreauii_group	0,51%	0,31%	0,79%
Butyricicoccus	0,49%	0,33%	0,80%
Lachnospira	0,49%	0,18%	0,80%
NK4A214_group	0,46%	0,19%	0,92%
Romboutsia	0,43%	0,14%	1,19%
[Eubacterium]_eligens_group	0,38%	0,15%	0,78%
Incertae_Sedis	0,37%	0,20%	0,82%
Clostridia_UCG-014 Order	0,36%	0,02%	1,31%
Erysipelotrichaceae_UCG-003	0,33%	0,08%	0,87%
[Eubacterium]_ventriosum_group	0,30%	0,14%	0,46%
Monoglobus	0,30%	0,13%	0,56%
Oscillospiraceae Family	0,24%	0,00%	0,54%
Clostridium_sensu_stricto_1	0,24%	0,04%	1,02%
CAG-56	0,21%	0,02%	0,39%
Holdemanella	0,21%	0,00%	2,08%
Lachnospiraceae_FCS020_group	0,19%	0,10%	0,30%

Phascolarctobacterium	0,19%	0,00%	0,55%
Prevotella	0,18%	0,00%	5,12%
Sutterella	0,17%	0,04%	0,35%
Family_XIII_AD3011_group	0,15%	0,09%	0,27%
Colidextribacter	0,14%	0,05%	0,22%
Akkermansia	0,14%	0,00%	1,14%
Marvinbryantia	0,13%	0,07%	0,25%
Intestinimonas	0,12%	0,04%	0,30%
Lachnospiraceae_UCG-001	0,12%	0,03%	0,23%
Lachnospiraceae_UCG-004	0,10%	0,03%	0,19%
Intestinibacter	0,09%	0,01%	0,47%
Bilophila	0,08%	0,00%	0,12%
Eggerthellaceae Family	0,07%	0,00%	0,32%
Senegalimassilia	0,07%	0,00%	0,21%
[Eubacterium]_xylanophilum_group	0,07%	0,00%	0,20%
Escherichia-Shigella	0,06%	0,02%	0,61%
Family_XIII_UCG-001	0,06%	0,04%	0,09%
UCG-003	0,06%	0,01%	0,11%
Barnesiella	0,05%	0,00%	0,26%
Oscillibacter	0,05%	0,02%	0,12%
Ruminococcaceae Family	0,05%	0,01%	0,15%
Clostridia Class	0,05%	0,02%	0,19%
Dialister	0,04%	0,00%	2,00%
Lachnospiraceae_UCG-010	0,04%	0,00%	0,10%
Negativibacillus	0,03%	0,00%	0,10%
Adlercreutzia	0,03%	0,00%	0,07%
UBA1819	0,03%	0,01%	0,05%
Slackia	0,03%	0,00%	0,10%
[Eubacterium]_siraeum_group	0,03%	0,00%	0,11%
Actinomyces	0,03%	0,02%	0,05%
Methanobrevibacter	0,03%	0,00%	0,56%
Odoribacter	0,02%	0,01%	0,05%
UCG-010 Family	0,02%	0,00%	0,07%
GCA-900066575	0,02%	0,00%	0,05%
Turicibacter	0,02%	0,00%	0,09%
Enterorhabdus	0,01%	0,00%	0,23%
Christensenellaceae Family	0,01%	0,00%	0,13%
Flavonifractor	0,01%	0,00%	0,04%
DTU089	0,01%	0,00%	0,03%
Candidatus_Soleaferrea	0,01%	0,00%	0,02%
[Eubacterium]_brachy_group	0,01%	0,00%	0,03%
Coriobacteriales_Incertae_Sedis Family	0,01%	0,00%	0,08%
Erysipelatoclostridium	0,01%	0,00%	0,04%
Parasutterella	0,01%	0,00%	0,02%
Terrisporobacter	0,00%	0,00%	0,07%
Coprobacter	0,00%	0,00%	0,03%

CAG-352	0,00%	0,00%	0,09%
[Eubacterium]_ruminantium_group	0,00%	0,00%	0,15%
[Ruminococcus]_gnavus_group	0,00%	0,00%	0,04%
Catenibacterium	0,00%	0,00%	0,01%
Succinivibrio	0,00%	0,00%	0,00%
Paraprevotella	0,00%	0,00%	0,36%
Solobacterium	0,00%	0,00%	0,00%
Muribaculaceae Family	0,00%	0,00%	0,00%
Alloprevotella	0,00%	0,00%	0,00%
Mogibacterium	0,00%	0,00%	0,00%
Klebsiella	0,00%	0,00%	0,00%
Megasphaera	0,00%	0,00%	0,00%
Acidaminococcus	0,00%	0,00%	0,00%
RF39 Order	0,00%	0,00%	0,08%
Prevotellaceae_NK3B31_group	0,00%	0,00%	0,00%
Haemophilus	0,00%	0,00%	0,03%
Mitsuokella	0,00%	0,00%	0,00%
Tyzzerella	0,00%	0,00%	0,00%
Desulfovibrio	0,00%	0,00%	0,25%
Lachnospiraceae_AC2044_group	0,00%	0,00%	0,01%
Megamonas	0,00%	0,00%	0,00%
Olsenella	0,00%	0,00%	0,05%
Peptococcus	0,00%	0,00%	0,00%
Butyricicoccaceae Family	0,00%	0,00%	0,00%
Bacteroidales Order	0,00%	0,00%	0,00%
Lactobacillus	0,00%	0,00%	0,00%
Rikenellaceae_RC9_gut_group	0,00%	0,00%	0,00%
Howardella	0,00%	0,00%	0,06%
Eggerthella	0,00%	0,00%	0,02%
Lactococcus	0,00%	0,00%	0,01%
Erysipelotrichaceae Family	0,00%	0,00%	0,04%
Rhodospirillales Order	0,00%	0,00%	0,03%
Gastranaerophilales Order	0,00%	0,00%	0,00%
Frisingicoccus	0,00%	0,00%	0,00%
Izemoplasmatales Order	0,00%	0,00%	0,00%
Faecalitalea	0,00%	0,00%	0,00%
Sellimonas	0,00%	0,00%	0,00%
Prevotellaceae Family	0,00%	0,00%	0,00%
Allisonella	0,00%	0,00%	0,02%
Shuttleworthia	0,00%	0,00%	0,00%
Barnesiellaceae Family	0,00%	0,00%	0,00%
Atopobiaceae Family	0,00%	0,00%	0,00%
Angelakisella	0,00%	0,00%	0,02%
Fournierella	0,00%	0,00%	0,03%
Oscillospira	0,00%	0,00%	0,02%
Veillonella	0,00%	0,00%	0,01%

Epulopiscium	0,00%	0,00%	0,00%
UCG-009	0,00%	0,00%	0,02%
Methanosphaera	0,00%	0,00%	0,00%
